# Metabolic rewiring at the obesity–estrogen–polyamine axis in endometrial cancer: emerging mechanisms and translational opportunities

**DOI:** 10.3389/fcell.2026.1954981

**Published:** 2026-10-08

**Authors:** Daniel Simancas-Racines, Dolores Jima Gavilanes, Andri Matos, Gabriele Davide Bigoni-Ordóñez, Claudia Reytor-González

**Affiliations:** 1 Center for Evidence Ecosystems, Implementation Science, and Decision-Making (CIDES), Facultad de Ciencias de la Salud y Bienestar Humano, Universidad Tecnológica Indoamérica, Ambato, Ecuador; 2 Escuela de Medicina, Universidad Espíritu Santo, Samborondón, Ecuador; 3 School of Allied Health, Eastwick College, Ramsey, NJ, United States; 4 Carrera de Laboratorio Clínico, Facultad de Ciencias Médicas, Universidad de Cuenca, Cuenca, Ecuador; 5 Grupo de Investigación en Salud Sexual y Reproductiva, Departamento de Investigación en Salud Pública, Universidad de Cuenca, Cuenca, Ecuador

**Keywords:** endometrial cancer, estrogen signaling, insulin resistance, metabolic syndrome, obesity, ornithine decarboxylase 1, polyamine metabolism, tumor microenvironment

## Abstract

Endometrial cancer is one of the gynecologic malignancies most strongly associated with obesity, yet the biological basis of this association extends beyond excess adiposity as a risk factor. Obesity reshapes the endometrial ecosystem through peripheral estrogen excess, progesterone imbalance, insulin resistance, adipokine dysregulation, chronic inflammation, and activation of anabolic signaling pathways. These mechanisms converge on metabolic programs that support proliferation, invasion, immune remodeling, and treatment resistance. Polyamine metabolism is emerging as a potentially relevant but still incompletely validated layer of this biology. Ornithine decarboxylase 1, the rate-limiting enzyme in polyamine biosynthesis, is overexpressed in aggressive endometrial cancers and has been associated with recurrence and poor survival. Recent evidence further suggests that metabolic syndrome-associated oleic acid may stabilize homeobox B9, protect ornithine decarboxylase 1 from degradation, increase polyamine accumulation, and promote tumor progression. This Mini Review critically examines the obesity–estrogen–polyamine axis as an integrative framework linking endocrine dysfunction, inflammatory remodeling, lipid signaling, and proliferative metabolic rewiring in endometrial cancer. It also discusses biomarker opportunities, therapeutic vulnerabilities, and the translational gaps that must be addressed before this axis can inform precision oncology.

## 1 Introduction

Endometrial cancer (EC) is commonly framed as a hormone-dependent malignancy, but this description is now insufficient. Obesity is a well-established and modifiable risk factor for EC, and epidemiological evidence supports a dose-dependent association between increasing body mass index (BMI) and disease incidence (Ordeanu et al., 2025; Mu et al., 2012; Kliemann et al., 2022; Chapela et al., 2025). However, BMI is an imprecise biological proxy. It does not capture visceral adiposity, adipose inflammation, insulin resistance, lipid dysfunction, or metabolic health status, all of which may shape tumor behavior (Simancas-Racines et al., 2025; Berstein et al., 2015; Mauland et al., 2017). Therefore, the central question is no longer whether obesity increases EC risk, but how obesity-associated endocrine and metabolic dysfunction is translated into tumor biology.

The classical model emphasizes estrogen excess. In obesity, adipose tissue expresses aromatase and converts androgens into estrogens, especially in postmenopausal women (Ordeanu et al., 2025; Selmi and La Marca, 2023). This increases unopposed estrogen exposure and promotes endometrial proliferation, particularly when progesterone signaling is deficient or impaired (Selmi and La Marca, 2023; Kim et al., 2013). Yet estrogen signaling does not operate in isolation. Insulin resistance and compensatory hyperinsulinemia activate phosphoinositide 3-kinase/protein kinase B/mechanistic target of rapamycin (PI3K/AKT/mTOR) and mitogen-activated protein kinase/extracellular signal-regulated kinase (MAPK/ERK) pathways, while also increasing estrogen bioavailability by reducing sex hormone-binding globulin (Gunter et al., 2008; Słabuszewska-Jóźwiak et al., 2022; Dashti et al., 2021; Pavlidou and Vlahos, 2014). This creates a biological setting in which endocrine and metabolic signals reinforce each other.

Recent mechanistic evidence supports viewing EC as an endocrine malignancy shaped by extensive metabolic rewiring. Estrogen signaling, insulin-like growth factor 1 (IGF-1) signaling, adipokine imbalance, chronic inflammation, lipid dysregulation, and tumor-intrinsic genomic alterations converge on anabolic and survival pathways (Raglan et al., 2020; Zhang et al., 2025; Catalano et al., 2009; Shen et al., 2024; Borghi et al., 2018; Han and Yuan, 2026; Tian et al., 2019; Grosso et al., 2022). Within this network, polyamine metabolism has emerged as a candidate node linking proliferative signaling with metabolic adaptation. Ornithine decarboxylase 1 (ODC1), the rate-limiting enzyme in polyamine biosynthesis, is upregulated in aggressive EC and is regulated by established oncogenic and metabolic inputs, including MYC and PI3K/AKT/mTOR signaling, while emerging evidence suggests additional regulation through the oleic acid–homeobox B9 (HOXB9) axis (Kim et al., 2017; Kim et al., 2022; Zhai et al., 2026). This review discusses how obesity-associated endocrine, inflammatory, lipid, and metabolic alterations may converge on polyamine metabolism in EC, while critically appraising the strength and limitations of the available evidence.

## 2 Methods

A focused literature search was conducted in MEDLINE/PubMed, Scopus, and the Cochrane Library from database inception through 16 March 2026, using controlled vocabulary, where available, and free-text terms adapted to each database. Search terms included combinations of “endometrial cancer,” “obesity,” “metabolic syndrome,” “estrogen,” “progesterone,” “insulin resistance,” “adipokines,” “inflammation,” “tumor microenvironment,” “lipid metabolism,” “oleic acid,” “polyamine metabolism,” “ornithine decarboxylase,” “ODC1,” and “HOXB9.” Studies were considered eligible when they provided clinical, translational, or mechanistic evidence directly relevant to the endocrine, metabolic, inflammatory, or polyamine pathways addressed in this review; studies without direct relevance to these mechanisms were excluded. Two reviewers independently screened titles and abstracts and subsequently assessed potentially eligible articles in full text, with disagreements resolved by a third reviewer. Reference lists of included articles were also screened for additional relevant studies. Priority was given to recent endometrial cancer–specific evidence, while seminal studies and mechanistically relevant experimental evidence were retained when necessary to establish biological context.

## 3 Obesity-associated endocrine and metabolic reprogramming in endometrial cancer

### 3.1 Estrogen excess and insulin-mediated anabolic signaling

Obesity promotes EC through interdependent endocrine and metabolic mechanisms rather than through one dominant pathway. Adipose-derived aromatase increases peripheral estrogen production, especially after menopause, and this favors estrogen receptor alpha-mediated endometrial proliferation when progesterone opposition is insufficient (Ordeanu et al., 2025; Selmi and La Marca, 2023). Loss or impairment of progesterone receptor (PR) signaling is particularly relevant because PR normally restrains estrogen-driven proliferation, and PR loss has been associated with higher tumor grade, hormone resistance, and worse outcomes (Kim et al., 2013; Qi et al., 2026). Thus, progesterone resistance should be considered a core component rather than a secondary feature of obesity-associated endometrial carcinogenesis.

Insulin resistance adds an anabolic signaling layer to this endocrine context. Hyperinsulinemia activates PI3K/AKT/mTOR and MAPK/ERK signaling, thereby promoting proliferation and survival in endometrial cells (Mu et al., 2012; Słabuszewska-Jóźwiak et al., 2022; Dashti et al., 2021; Raglan et al., 2020; Zhang et al., 2025). Insulin resistance may also increase estrogen sensitivity through epigenetic mechanisms, including TET1-mediated upregulation of G protein-coupled estrogen receptor, which enhances estrogen-driven PI3K/AKT activation (Lv et al., 2017; Cignarella et al., 2023). This is important because it shifts the model from simple “estrogen excess” to reciprocal endocrine-metabolic amplification.

The PI3K/AKT/mTOR pathway is a major convergence point. It is activated by insulin and IGF-1 signaling, but it is also reinforced by tumor-intrinsic alterations, particularly *PTEN* loss and *PIK3CA* mutations, as well as less consistently characterized alterations involving *PIK3CB* and protein phosphatase 2A regulation (Asami et al., 2023; Passarelli et al., 2024; Tyrakis et al., 2025). Moreover, compensatory activation of receptor tyrosine kinases and MAPK/ERK signaling can limit the effectiveness of PI3K/AKT/mTOR inhibition (Paplomata and O’Regan, 2014; Aslan et al., 2018). Although biologically coherent, this evidence is not yet clinically definitive. Much of the mechanistic support comes from cell lines, animal models, molecular profiling, and selected clinical cohorts rather than prospective trials stratified by metabolic phenotype.

Lipid abnormalities further complicate this network. Dyslipidemia, including elevated triglycerides and reduced high-density lipoprotein cholesterol, may contribute to oxidative stress, DNA damage, and genomic instability (Włodarczyk and Nowicka, 2019; Wang et al., 2024; Lou et al., 2024; Liu et al., 2024). EC also shows alterations in lipid biosynthesis, fatty acid oxidation, sphingolipid metabolism, glucose metabolism, amino acid metabolism, and one-carbon metabolism (Wang et al., 2024; Yan et al., 2024; Mohd et al., 2025; Fang et al., 2024). This breadth of metabolic remodeling suggests that single-pathway explanations are probably inadequate. It also means that therapeutic targeting will require patient selection, because not all obese patients, and not all EC tumors, are metabolically equivalent (Hishinuma et al., 2023; Pietkiewicz et al., 2025).

### 3.2 Adipokines, inflammation, and tumor microenvironment remodeling

Dysfunctional adipose tissue behaves as a chronic inflammatory organ. Although direct evidence from endometrial tumor tissue remains limited, inflammatory remodeling mechanisms described in obesity and related gynecologic conditions, including NLRP3 inflammasome activation, Toll-like receptor 4/nuclear factor kappa B (NF-κB) signaling, endoplasmic reticulum stress, and increased cytokine release, are biologically plausible contributors in obesity-associated EC (Engin, 2024; Wani et al., 2021; Jiang et al., 2025; Zhang et al., 2021). These pathways may promote proliferation, angiogenesis, immune dysregulation, and metabolic stress; however, their specific contribution within the endometrial tumor microenvironment remains insufficiently characterized (Xu H. Y. et al., 2023; Ha et al., 2021; Hu et al., 2021; Vitseva et al., 2008; Velloso et al., 2015). Importantly, inflammation is not merely a background condition but an active driver of tumor microenvironment (TME) remodeling.

The opposing actions of leptin and adiponectin illustrate this adipokine imbalance. Leptin is increased in obesity and activates Janus kinase 2/signal transducer and activator of transcription 3, PI3K/AKT/mTOR, and MAPK/ERK signaling (Catalano et al., 2009; Shen et al., 2024; Wu et al., 2012; Olea-Flores et al., 2020). Through these pathways, leptin promotes proliferation, epithelial–mesenchymal transition, invasion, and angiogenesis (Catalano et al., 2009; Shen et al., 2024; Olea-Flores et al., 2020; Gao et al., 2009). Adiponectin generally has opposing effects through adenosine monophosphate-activated protein kinase and related metabolic pathways, reducing proliferative and inflammatory signaling (Ray et al., 2022; Bieńkiewicz et al., 2012; Moon et al., 2011; Jin et al., 2021; Zhang et al., 2015). The leptin-to-adiponectin ratio may therefore better reflect metabolic risk than either marker alone, but its clinical use remains limited by assay variability, confounding by insulin resistance, and heterogeneity in patient populations (Ray et al., 2022; Ellis et al., 2020; Ashizawa et al., 2010; Zhu et al., 2023; Turan et al., 2022).

Cytokine networks provide another amplification loop. Interleukin-6 activates signal transducer and activator of transcription 3 (STAT3), while tumor necrosis factor-alpha promotes NF-κB and MAPK/ERK signaling (Yamauchi et al., 2004; Bersinger et al., 2011; Che et al., 2014; Yu et al., 2014; Zeng et al., 2022). NF-κB and STAT3 can cooperate to regulate anti-apoptotic, proliferative, proangiogenic, and immune-modulatory genes, including *BCL2, BCL2L1, CCND1, MYC, PTGS2, BIRC5, MMP2, and HIF1A* (Puar et al., 2018; Yang et al., 2010; Zdrojkowski et al., 2023; Chen et al., 2007; Grivennikov and Karin, 2010). This provides a plausible molecular link between obesity-associated inflammation and aggressive tumor behavior. However, the evidence remains uneven. Some associations are supported by clinical samples and meta-analyses, whereas many causal claims rely on preclinical systems that do not fully reproduce the human obesity-associated TME (Ray et al., 2022; Naqvi et al., 2022; Iyengar et al., 2016).

At the cellular level, obesity may alter immune and stromal architecture. Reported changes include expansion of regulatory T cells, M2-like macrophage polarization, impaired CD8-positive T-cell function, stromal activation, extracellular matrix remodeling, and adipocyte-derived extracellular vesicle signaling (Jeong and Kim, 2026; Giatromanolaki et al., 2008; Artuyants et al., 2024; Kolben et al., 2022). These findings are important but still fragmented. Longitudinal human studies are scarce, and diabetes, menopausal status, treatment exposure, and molecular subtype are often insufficiently integrated. This limits the ability to determine whether inflammatory remodeling is a driver of progression, a marker of metabolic disease, or both.

## 4 Polyamine metabolism as an emerging metabolic node

### 4.1 ODC1 and polyamine biosynthesis in proliferative signaling

Polyamines, mainly putrescine, spermidine, and spermine, are required for nucleic acid stabilization, chromatin organization, translation, cell-cycle progression, and cellular adaptation to stress (Kim et al., 2017; Kim et al., 2022; Pegg, 2006; Choi et al., 2016; Basuroy and Gerner, 2006). ODC1 catalyzes the first and rate-limiting step of polyamine biosynthesis by converting ornithine to putrescine (Kim et al., 2017; Coni et al., 2023; Chanda and Ganguly, 1995). In cancer, this pathway is frequently upregulated to support proliferation and survival (Kim et al., 2017; Choi et al., 2016; Ye et al., 2019).

In EC, *ODC1* is overexpressed in aggressive tumors, including non-endometrioid, high-grade, and copy-number-high cancers (Kim et al., 2017). High *ODC1* expression has been associated with worse overall survival, increased recurrence, and shorter recurrence-free survival in patient cohorts (Kim et al., 2017). Tumor tissues also show increased putrescine, spermidine, and spermine compared with normal endometrium (Kim et al., 2017; Kim et al., 2022; Chanda and Ganguly, 1995). These observations support the relevance of the pathway, but they do not yet prove clinical actionability. Although expression and metabolite data are promising, prospective validation and treatment-response studies remain limited (Basuroy and Gerner, 2006; Düz et al., 2022; Skorupa et al., 2021; Alexiou et al., 2017; Maddox et al., 1985).

ODC1 is regulated by both oncogenic and metabolic inputs. MYC directly increases *ODC1* transcription and supports polyamine flux (Kim et al., 2022; Shantz and Levin, 2007; Bachmann and Geerts, 2018). PI3K/AKT/mTOR signaling can increase *ODC1* expression and activity, and pathway inhibition can reduce ODC1 and polyamine levels in experimental models (Kim et al., 2022; Gadducci et al., 2008). ODC1 is also controlled post-translationally through antizyme-mediated degradation and antizyme inhibitor rescue (Kahana, 2016; Almrud et al., 2000; Kahana, 2009; Qiu et al., 2017). This multilayered regulation makes ODC1 biologically plausible as a convergence node. It also makes therapeutic targeting difficult, because tumors may compensate through transport, salvage, or parallel anabolic signaling.

Molecular subtype is likely to influence the biological relevance of polyamine rewiring. Analysis of The Cancer Genome Atlas data showed that *ODC1* is expressed across the four original molecular groups, *POLE* ultramutated, microsatellite instability hypermutated, copy-number low, and copy-number high, but is significantly more highly expressed in copy-number-high tumors (Kim et al., 2017). These groups broadly correspond to the current *POLE*-mutated, mismatch repair-deficient (MMRd), no specific molecular profile, and p53-abnormal classes, respectively. Copy-number-high/p53-abnormal tumors are characterized by frequent *TP53* alterations, whereas *PTEN* alterations are more characteristic of endometrioid molecular groups (Levine, 2013). *HOXB9* has been evaluated in endometrial cancer according to histological subtype and *TP53* mutation status; however, its expression across the four validated molecular classes has not been systematically established (Xu Y. et al., 2023). Obesity-associated mechanisms also appear unlikely to operate uniformly across molecular subtypes, as higher BMI has been particularly associated with no specific molecular profile tumors, with additional BMI-related heterogeneity reported within MMRd disease (Smith et al., 2024). *PTEN* loss may plausibly enhance PI3K/AKT/mTOR signaling and thereby favor ODC1-dependent polyamine synthesis, although a direct subtype-specific relationship remains unproven. Conversely, the enrichment of *ODC1* in copy-number-high tumors suggests that polyamine rewiring may also occur through *PTEN*-independent oncogenic mechanisms, including MYC-related signaling (Kim et al., 2017). Overall, these findings support molecularly stratified investigation of the obesity–polyamine axis rather than assuming uniform biological relevance across EC subtypes.

### 4.2 Lipid metabolism and obesity-associated polyamine rewiring

The connection between lipid excess and polyamine accumulation represents a particularly novel component of this framework. In metabolic syndrome-associated EC, oleic acid has been reported to stabilize HOXB9 by preventing PRAJA2-mediated degradation (Zhai et al., 2026; Casero et al., 2018). Stabilized HOXB9 has been reported to bind ODC1, compete with antizyme 1, protect ODC1 from degradation, and increase polyamine accumulation (Zhai et al., 2026; Casero et al., 2018). This emerging mechanism suggests a potential link between a metabolic syndrome-associated fatty acid and the post-translational stabilization of a polyamine biosynthetic enzyme.

This evidence provides the most specific, although still preliminary, mechanistic support for the proposed obesity–estrogen–polyamine framework. The model is no longer limited to “obesity increases estrogen” or “obesity increases inflammation.” It proposes that lipid signaling may stabilize a specific oncogenic-polyamine module that supports tumor growth and may influence treatment response (Zhai et al., 2026; Casero et al., 2018). However, the evidence should be interpreted cautiously. The oleic acid–HOXB9–ODC1 axis is best characterized in metabolic syndrome-associated EC, and its generalizability across EC molecular subtypes, menopausal groups, disease stages, and treatment contexts is not yet established (Borghi et al., 2018; Akkour et al., 2026; Yao et al., 2024). The structural basis of the HOXB9–ODC1 interaction and the epigenetic regulation of *HOXB9* remain incompletely characterized (Borghi et al., 2018; Akkour et al., 2026; Yao et al., 2024).

Polyamine biology also intersects with other metabolic processes. Polyamine catabolism through spermidine/spermine N1-acetyltransferase and spermine oxidase can alter acetyl-coenzyme A availability, reactive oxygen species production, fatty acid oxidation, and cellular energetics (Duranton et al., 2003; Wu et al., 2015). Spermidine-dependent eukaryotic translation initiation factor 5A hypusination supports translation elongation and tumor progression, and combined inhibition of ODC1 and hypusination has shown antitumor activity in EC models (Kim et al., 2022; Coni et al., 2023). These mechanisms are attractive, but they also reveal a therapeutic paradox: polyamines can support tumor growth, but under certain catabolic and oxidative conditions they may also contribute to ferroptotic vulnerability (Pignatti et al., 2004; Yerlikaya, 2026; Wu et al., 2022). Any therapeutic strategy must account for this context dependence.

Collectively, these findings support a hypothesis-generating model in which obesity-associated endocrine, inflammatory, insulin-dependent, and lipid-derived signals may converge on ODC1 regulation and polyamine accumulation, potentially contributing to endometrial cancer progression (Figure 1).

**FIGURE 1:**
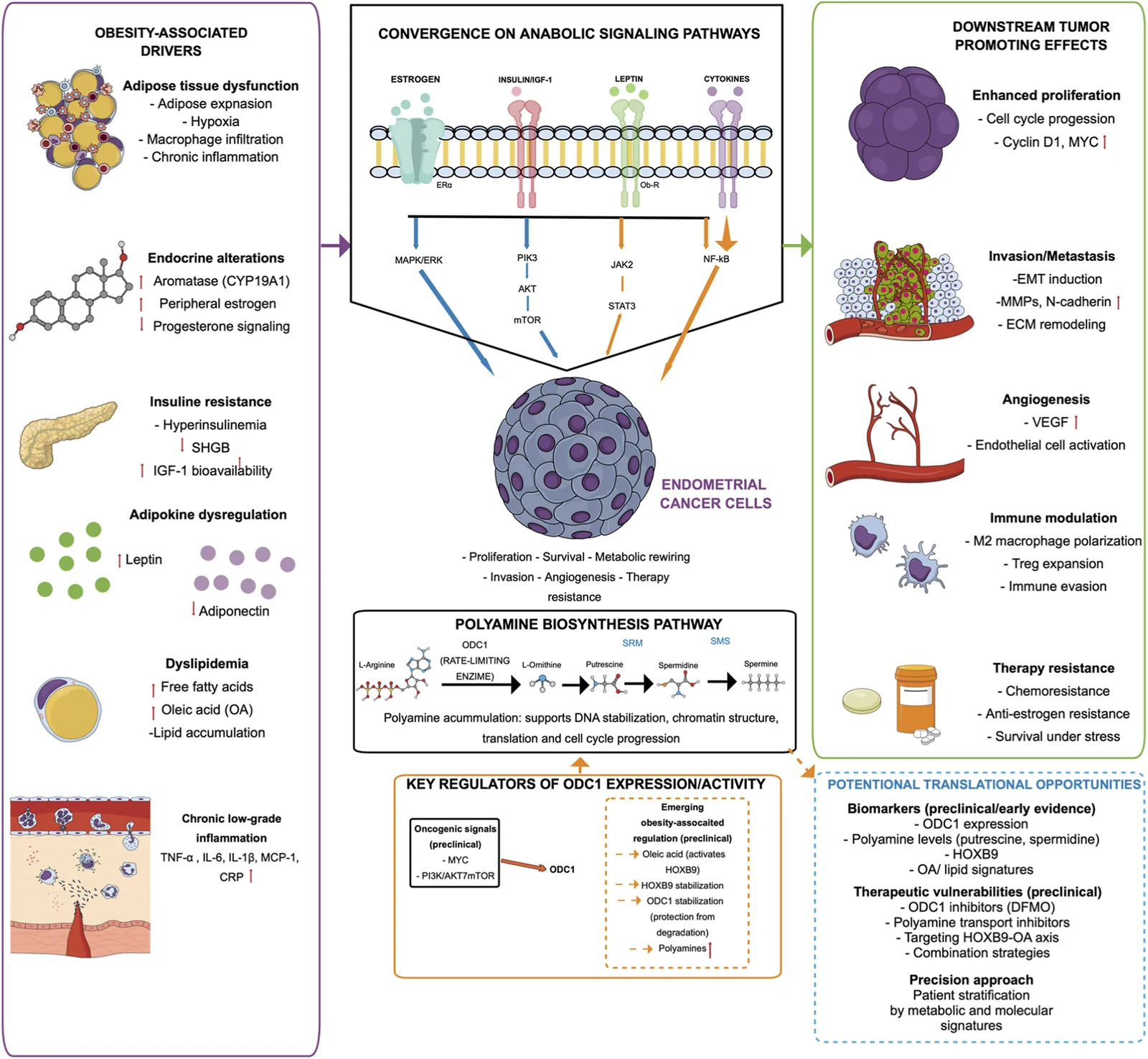
The obesity–estrogen–polyamine axis in endometrial cancer. Obesity-associated endocrine, metabolic, inflammatory, and lipid alterations activate ERα/MAPK–ERK, insulin–IGF-1/PI3K–AKT–mTOR, leptin/Ob-R–JAK2–STAT3, and cytokine/NF-κB signaling. These pathways may converge on ODC1-dependent polyamine biosynthesis and tumor-promoting phenotypes. Emerging evidence suggests that oleic acid-mediated HOXB9 stabilization may protect ODC1 from degradation, potentially increasing polyamine accumulation. Potential translational opportunities include ODC1 inhibition, polyamine transport blockade, and biomarker-guided therapeutic strategies. Solid arrows indicate established or comparatively well-validated biological relationships, whereas dashed arrows denote emerging or hypothesis-generating mechanisms. Blue arrows indicate relationships supported predominantly by human/clinical evidence, whereas orange arrows represent predominantly preclinical or mechanistic evidence. Abbreviations: ERα, estrogen receptor alpha; HOXB9, homeobox B9; IGF-1, insulin-like growth factor 1; JAK2, Janus kinase 2; MAPK/ERK, mitogen-activated protein kinase/extracellular signal-regulated kinase; NF-κB, nuclear factor kappa B; Ob-R, leptin receptor; ODC1, ornithine decarboxylase 1; PI3K/AKT/mTOR, phosphoinositide 3-kinase/protein kinase B/mechanistic target of rapamycin; STAT3, signal transducer and activator of transcription 3.

### 4.3 Translational implications, therapeutic vulnerabilities, and barriers to clinical translation

ODC1 and polyamine pathway activity may have biomarker potential; however, their clinical readiness remains low. *ODC1* expression may have prognostic value for recurrence risk, and polyamine pathway activity may help identify tumors with metabolic dependence (Kim et al., 2017; Rosario et al., 2023). However, quantitative polyamine metabolite data by tumor grade, molecular subtype, metabolic syndrome status, and treatment response are still sparse (Kim et al., 2017; Basuroy and Gerner, 2006; Düz et al., 2022; Skorupa et al., 2021; Deng et al., 2008). This is a major limitation. Without robust metabolomic validation, ODC1 may remain a biologically plausible marker without sufficient clinical utility.

Therapeutically, difluoromethylornithine (DFMO), an ODC1 inhibitor, is the most extensively studied strategy for targeting polyamine biosynthesis (Kim et al., 2017; Kim et al., 2022; Liu et al., 2025; Burns et al., 2009; Piha-Paul et al., 2025). In EC models, ODC1 inhibition reduces polyamine levels, cell viability, and tumor growth (Kim et al., 2017; Kim et al., 2022). However, these findings remain preclinical, and therapeutic efficacy has not yet been established in EC-specific clinical trials. An additional barrier is metabolic adaptation following ODC1 inhibition. Suppression of intracellular polyamine synthesis can promote compensatory uptake of extracellular polyamines, potentially reducing the effectiveness of DFMO monotherapy and providing a rationale for simultaneously targeting polyamine biosynthesis and transport (Casero et al., 2018; Burns et al., 2009; Raul, 2007). A recent phase I study combining the polyamine transport inhibitor AMXT 1501 with DFMO demonstrated feasibility and preliminary antitumor activity in advanced solid tumors, although it was not specific to EC (Piha-Paul et al., 2025). Treatment-related toxicity also requires consideration: gastrointestinal adverse events, including diarrhea, nausea, and vomiting, were frequently reported with the combination (Piha-Paul et al., 2025), while long-term DFMO exposure has been associated with increased hearing thresholds, supporting appropriate auditory monitoring (Chow et al., 2023).

A further translational challenge is the absence of validated patient-selection strategies. EC exhibits substantial metabolic heterogeneity across lipid, glucose, amino acid, pentose phosphate, one-carbon, and sphingolipid pathways (Yan et al., 2024; Fang et al., 2024; Srivastava and Vinod, 2023; Huang et al., 2023), while genomic alterations such as *PTEN* loss and *TP53* mutation may define subgroups with distinct metabolic vulnerabilities (Liu et al., 2024; Srivastava and Vinod, 2023; Inaba et al., 2005; Schultheis et al., 2016; Co et al., 2014). *ODC1* expression, polyamine abundance, HOXB9 activity, and metabolic syndrome status therefore remain candidate biomarkers rather than validated predictors of therapeutic response, while molecular subtype may provide an additional framework for patient stratification (Kim et al., 2017; Zhai et al., 2026; Rosario et al., 2023). Patient-derived organoids, spatial multi-omics, metabolomics, and machine learning-based subtyping may improve translation, but these approaches remain early and require standardization before clinical use (Liu et al., 2024; Van Nyen et al., 2018; Wu et al., 2025). Future clinical trials should incorporate prospective molecular and metabolic profiling together with pharmacodynamic assessment of polyamine pathway activity to identify responsive subgroups and distinguish prognostic associations from true predictive biomarkers. The future of this field should be biomarker-guided and mechanism-based rather than driven by pathway plausibility alone. The relative strength of evidence and translational readiness of the main components of the obesity–estrogen–polyamine axis is summarized in Table 1.

**TABLE 1:** Evidence strength and translational readiness of the obesity–estrogen–polyamine axis in endometrial cancer.

Axis component	Evidence base	Biological interpretation	Clinical translation maturity
Obesity–estrogen excess–progesterone imbalance	Epidemiological, endocrine, and clinical evidence	Established hormonal-risk axis for hormone-sensitive tumorigenesis	●●
Insulin resistance–PI3K/AKT/mTOR activation	Molecular, cellular, genomic, and clinical evidence	Central metabolic–oncogenic pathway associated with therapy resistance	●●
Adipokines, cytokines, and TME remodeling	Meta-analyses, cohorts, experimental models, and immune profiling	Inflammatory and immune-metabolic axis involved in invasion and angiogenesis	●–●●
ODC1 and polyamine biosynthesis	Transcriptomics, IHC, metabolomics, xenograft, cellular, and clinical correlation data	Emerging metabolic vulnerability associated with aggressive disease	●–●●
Oleic acid–HOXB9–ODC1 axis	Recent mechanistic and patient-derived evidence	Novel lipid–transcriptional–polyamine axis requiring validation	●

Abbreviations: EC, endometrial cancer; HOXB9, homeobox B9; IHC, immunohistochemistry; ODC1, ornithine decarboxylase 1; PI3K/AKT/mTOR, phosphoinositide 3-kinase/protein kinase B/mechanistic target of rapamycin pathway; TME, tumor microenvironment. Clinical translation maturity was classified using four domains: (1) evidence from human EC, cohorts, (2) prospective or independent validation, (3) biomarker standardization and reproducibility, and (4) EC-specific evidence supporting clinical implementation or therapeutic targeting. ●, low maturity: evidence is predominantly preclinical, exploratory, or associative, with absent or limited prospective validation and no established clinical implementation. ●●, moderate maturity: supported by convergent human and mechanistic evidence, but with incomplete prospective validation, biomarker standardization, or EC-specific therapeutic evidence. ●–●● indicates heterogeneous evidence across these domains.

## 5 Discussion

The proposed obesity–estrogen–polyamine axis provides an integrative framework connecting four interconnected domains of EC biology that are often discussed separately: endocrine carcinogenesis, metabolic dysfunction, obesity-associated inflammation, and polyamine rewiring. Importantly, the strength of evidence supporting these components differs substantially. The obesity–estrogen–progesterone axis and insulin-related signaling are supported by established epidemiological, clinical, and molecular evidence, whereas ODC1-dependent polyamine rewiring represents an emerging mechanism and the oleic acid–HOXB9–ODC1 pathway should currently be considered hypothesis-generating. The endocrine layer encompasses estrogen excess, PR impairment, and hormone-driven proliferation (Ordeanu et al., 2025; Selmi and La Marca, 2023; Kim et al., 2013; Carlson et al., 2012). The metabolic layer encompasses insulin resistance, compensatory hyperinsulinemia, PI3K/AKT/mTOR activation, and broad alterations in lipid and intermediary metabolism (Mu et al., 2012; Raglan et al., 2020; Tian et al., 2019; Asami et al., 2023; Wang et al., 2024; Liu et al., 2024; Yan et al., 2024). The inflammatory layer involves adipokine imbalance, cytokine signaling, immune remodeling, and TME dysfunction (Catalano et al., 2009; Shen et al., 2024; Ray et al., 2022; Ellis et al., 2020; Che et al., 2014; Zeng et al., 2022; Naqvi et al., 2022; Giatromanolaki et al., 2008; Artuyants et al., 2024; Kolben et al., 2022). Finally, the polyamine layer offers a candidate effector pathway through which proliferative and metabolic signals may support tumor growth, chromatin adaptation, translation, and therapy resistance (Kim et al., 2017; Kim et al., 2022; Zhai et al., 2026; Pegg, 2006). Because obesity-associated inflammation and metabolic dysfunction are potentially modifiable, nutritional interventions may represent complementary strategies for attenuating the systemic conditions that support obesity-related carcinogenesis (Chapela et al., 2026; Muscogiuri et al., 2022; Kantilafti et al., 2025).

The strongest aspect of the evidence is biological plausibility across multiple levels of analysis. Epidemiological and clinical studies support the obesity–EC association (Ordeanu et al., 2025; Mu et al., 2012; Kliemann et al., 2022). Molecular and pathway studies support crosstalk between estrogen, insulin resistance, and PI3K/AKT/mTOR activation (Mu et al., 2012; Guo et al., 2006; Slomovitz and Coleman, 2012; Usman and Volpi, 2018). Preclinical and patient-derived data support *ODC1* overexpression and polyamine accumulation in aggressive EC (Kim et al., 2017; Kim et al., 2022; Chanda and Ganguly, 1995). The oleic acid–HOXB9–ODC1 mechanism provides preliminary mechanistic evidence suggesting a potential link between metabolic syndrome and polyamine accumulation (Zhai et al., 2026; Casero et al., 2018). However, the strength of this evidence is not uniform: the obesity–EC association is well established, ODC1 involvement is emerging, and the oleic acid–HOXB9–ODC1 mechanism currently rests largely on a single mechanistic study that requires independent replication.

The weakest aspect is clinical validation. Much of the evidence supporting polyamine rewiring is preclinical, associative, or based on selected cohorts. There are limited prospective data showing that polyamine-related biomarkers predict treatment response, recurrence, or survival beyond established clinicopathological and molecular variables (Basuroy and Gerner, 2006; Skorupa et al., 2021). There is also insufficient evidence to determine whether metabolic syndrome, oleic acid exposure, HOXB9 stabilization, and ODC1 activity define a reproducible clinical subgroup across EC molecular classes (Zhai et al., 2026; Tyrakis et al., 2025; Mohd et al., 2025; Casero et al., 2018; Raul, 2007). This matters because mechanistic elegance does not equal clinical utility. Therefore, these mechanistic findings should be interpreted as hypothesis-generating rather than evidence of an established causal pathway until validated in clinically annotated cohorts.

Future research should prioritize validation over expansion. First, ODC1, HOXB9, polyamine metabolites, estrogen metabolites, adipokines, and inflammatory markers should be mapped across molecular subtypes, menopausal status, BMI categories, insulin resistance, lipid profiles, and diabetes status (Liu et al., 2024; Srivastava and Vinod, 2023; Wu et al., 2025). Second, longitudinal cohorts should determine whether polyamine rewiring precedes progression or merely reflects aggressive tumor biology (Basuroy and Gerner, 2006; Papatla et al., 2016; Kryukova et al., 2025). Third, patient-derived organoids and spatial multi-omics should test whether polyamine dependence is tumor-cell intrinsic or shaped by stromal, immune, and adipose-derived signals (Iyengar et al., 2016; Van Nyen et al., 2018). Finally, clinical trials of polyamine-targeted strategies should be biomarker-guided and rationally combined with endocrine, PI3K/AKT/mTOR, immune, or lipid-metabolic interventions (Casero et al., 2018; Soberanis Pina and Lheureux, 2024; Barra et al., 2019).

In conclusion, EC may be conceptualized as a metabolically rewired endocrine malignancy. The obesity–estrogen–polyamine axis does not replace the classical estrogen-driven model; it refines it by positioning polyamine metabolism as a candidate downstream effector of endocrine, inflammatory, lipid, and oncogenic signaling. However, this axis should currently be regarded as an integrative framework in which established endocrine and metabolic mechanisms coexist with emerging and hypothesis-generating polyamine-related pathways, rather than as a fully established causal pathway. Its translational value will depend on whether ODC1, HOXB9, and polyamine metabolites can improve patient stratification, identify therapeutic vulnerabilities, and guide rational combinations beyond current molecular classification.

## Glossary

### Abbreviations

BMI: body mass index
DFMO: difluoromethylornithine
EC: endometrial cancer
ERα: estrogen receptor alpha
HOXB9: homeobox B9
IGF-1: insulin-like growth factor 1
IHC: immunohistochemistry
JAK2: Janus kinase 2
MAPK/ERK: mitogen-activated protein kinase/extracellular signal-regulated kinase
MMRd: mismatch repair-deficient
NF-κB: nuclear factor kappa B
Ob-R: leptin receptor
ODC1: ornithine decarboxylase 1
PI3K/AKT/mTOR: phosphoinositide 3-kinase/protein kinase B/mechanistic target of rapamycin
PR: progesterone receptor
STAT3: signal transducer and activator of transcription 3
TME: tumor microenvironment

### Gene symbols and definitions

*BCL2*, BCL2: apoptosis regulator
*BCL2L1*, BCL2: like 1
*BIRC5*: baculoviral IAP repeat containing 5
*CCND1*: cyclin D1
*HIF1A*: hypoxia inducible factor 1 subunit alpha
*MMP2*: matrix metallopeptidase 2
*MYC*, MYC: proto-oncogene, bHLH transcription factor
*NLRP3*, NLR: family pyrin domain containing 3
*PIK3CA*: phosphatidylinositol-4,5-bisphosphate 3-kinase catalytic subunit alpha
*PIK3CB*: phosphatidylinositol-4,5-bisphosphate 3-kinase catalytic subunit beta
*POLE*: DNA polymerase epsilon, catalytic subunit
*PRAJA2*: praja ring finger ubiquitin ligase 2
*PTEN*: phosphatase and tensin homolog
*PTGS2*: prostaglandin-endoperoxide synthase 2
*TET1*: TET methylcytosine dioxygenase 1
*TP53*: tumor protein p53
